# Inducing Mechanical Stimuli to Tissues Grown on a Magnetic Gel Allows Deconvoluting the Forces Leading to Traumatic Brain Injury

**DOI:** 10.1089/neur.2023.0026

**Published:** 2023-08-23

**Authors:** Luise Schlotterose, Megane Beldjilali-Labro, Mario Hagel, Moran Yadid, Carina Flaxer, Eli Flaxer, A. Ronny Barnea, Kirsten Hattermann, Esther Shohami, Yael Leichtmann-Bardoogo, Ben M. Maoz

**Affiliations:** ^1^ Institute of Anatomy, Kiel University, Kiel, Germany.; ^2^ Department of Biomedical Engineering, Tel Aviv University, Tel Aviv, Israel.; ^3^ The Azrieli Faculty of Medicine, Bar Ilan University, Safed, Israel.; ^4^ AFEKA–Tel-Aviv Academic College of Engineering, Tel-Aviv, Israel.; ^5^ Institute for Drug Research, The Hebrew University of Jerusalem, Jerusalem, Israel.; ^6^ Sagol School of Neuroscience, Tel Aviv University, Tel Aviv, Israel.; ^7^ The Center for Nanoscience and Nanotechnology, Tel Aviv University, Tel Aviv, Israel.; ^8^ Sagol Center for Regenerative Medicine, Tel Aviv University, Tel Aviv, Israel.

**Keywords:** acceleration-deacceleration, *in vitro* models, *in vitro* TBI, neurovascular unit, tension and compression

## Abstract

Traumatic brain injury (TBI), which is characterized by damage to the brain resulting from a sudden traumatic event, is a major cause of death and disability worldwide. It has short- and long-term effects, including neuroinflammation, cognitive deficits, and depression. TBI consists of multiple steps that may sometimes have opposing effects or mechanisms, making it challenging to investigate and translate new knowledge into effective therapies. In order to better understand and address the underlying mechanisms of TBI, we have developed an *in vitro* platform that allows dynamic simulation of TBI conditions by applying external magnetic forces to induce acceleration and deceleration injury, which is often observed in human TBI. Endothelial and neuron-like cells were successfully grown on magnetic gels and applied to the platform. Both cell types showed an instant response to the TBI model, but the endothelial cells were able to recover quickly—in contrast to the neuron-like cells. In conclusion, the presented *in vitro* model mimics the mechanical processes of acceleration/deceleration injury involved in TBI and will be a valuable resource for further research on brain injury.

## Introduction

Traumatic brain injury (TBI) is a significant burden to healthcare worldwide, with ∼1.7 million cases occurring annually in the United States. It is the leading cause of death in the adolescent population.^1^ TBI is generally described as an abrupt head movement in response to mechanical forces, which are often followed by damage to the brain tissue. TBI is categorized from mild to severe and has a range of short- and long-term effects on a person's quality of life, expressed by physical, cognitive, and emotional functioning.^2^ Whereas TBI can be associated with focal skull fractures, ∼40% of all TBI patients admitted to hospitals are non-focal injuries and are usually identified by diffuse axonal injury (DAI).^3,4^ This type of injury is commonly a result of inertial-induced loads that arise when the skull is accelerated while the brain mass continues its motion relative to the skull, which produces strains in the brain tissue.^5^

A major challenge in addressing TBI is the complex physio- and pathophysiological mechanisms associated with TBI. Indeed, the pathophysiology of TBI involves a complex interplay of harmful (excitotoxicity, mitochondrial dysfunction, oxidative stress, lipid peroxidation, etc.) and protective factors (neurogenesis, gliogenesis, angiogenesis, synaptic plasticity, and axonal sprouting) that determine the extent and severity of the injury, as well as the recovery.^6^ Despite significant progress in modeling^7,8^ and understanding the complex molecular mechanisms underlying TBI, such as activation of the inflammatory response,^9,10^ disruption of the blood–brain barrier,^11^ and release of excitatory neurotransmitters,^12^ there is still a gap in translating this knowledge into clinical practice and effective therapy.

The existence of a wide range of *in vitro* TBI models has been well documented.^13,14^ In the context of penetrating injuries, one approach is to perform a transection, which directly injures multiple cells while exposing surrounding cells to secondary effects. However, this method, though easily scalable and suitable for high-throughput screenings, is limited in its ability to model a small percentage of clinically relevant brain injuries.

On the other hand, to model non-penetrating brain injuries, various forces applied to cells or tissues have been established. Compression, shear strains, hydrostatic pressure, and stretch models are examples of such forces. These models offer the advantage of being easily controllable, widely used, and well characterized.^13,15–19^

**FIG. 1.**
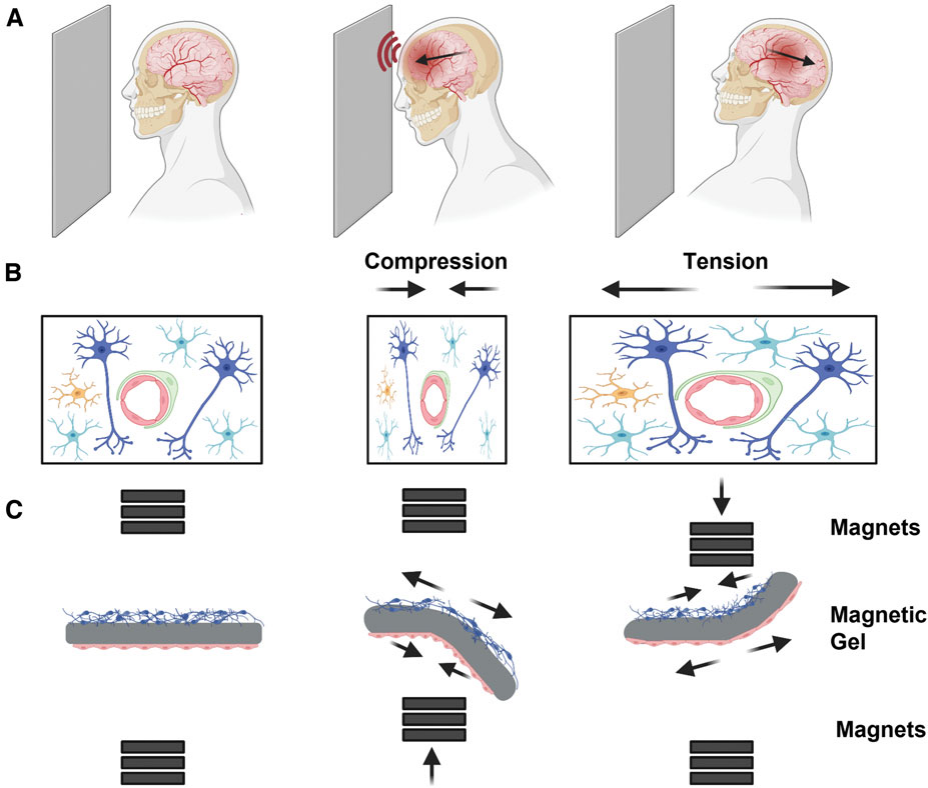
Schematic experimental description. (**A**) Acceleration-deceleration injury illustration. During the acceleration movement the brain tissue is compressed inside the skull and then put under tension in the following deceleration movement (**B**) Cellular response to TBI during acceleration and deceleration. Blue neurons, cyan: astrocytes, yellow: microglia cells, green: pericyte, red: endothelial cells. (**C**) The DTL system allows, by controlling the movement of the magnets, to bend the magnetic hydrogel, which causes a tension and compression effect on the cell. DTL, dynamic tissue-loading.

However, it is worth noting that these models have generally received little attention regarding the effects of isolated acceleration and deceleration injuries and their subsequent pathophysiology. As a result, coup and contrecoup injuries, which are clinically highly relevant in terms of DAI, are typically not adequately represented in these models^20^ (Fig. 1A).

This process imposes opposing forces on brain tissue, specifically on the neurovascular unit (NVU), which consists of brain vasculature and brain parenchyma (Fig 1B).^21^ It is difficult to separate these two mechanical processes *in vivo*, given that the brain is a viscoelastic material that behaves, to some extent, like a spring when compressed. Therefore, investigating TBI requires a comprehensive and multi-disciplinary approach to address the various physio- and pathophysiological mechanisms involved. Currently, computational simulations are the main tool to overcome this challenge. They allow the dynamic forces on tissue to be monitored, but there are no experimental tools that allow the separation of these processes. Moreover, *in silico* models do not allow to study the effects of drugs or environmental factors that affect outcome in TBI patients.

In this study, we present an *in vitro* platform that enables dynamic tissue loading (DTL) by applying external magnetic forces over a magnetic hydrogel, which represents brain tissue, and inducing complex TBI conditions, by rapid angular/rotational acceleration-deceleration, with no contact injuries (Fig. 1C). Once characterized, we demonstrated the platform's ability to induce injury on different cell types in mono- or cocultures on either side of a magnetic hydrogel.

## Methods

### Magnetic nanoparticle synthesis

Synthesis of magnetic nanoparticles (MNPs) was carried out as previously published by Fied and colleagues.^22^ Briefly, to form magnetic Fe_3_O_4_, FeCl_2_·4 H_2_O and FeCl_3_ were mixed at a 1:2 ratio in a basic solution of hydroxide ammonium at 85°C for 2 h under nitrogen atmosphere. After multiple magnetic decantations, to wash the MNPs, a hydrophilic coating of meso-2,3-dimercaptosuccinic acid (DMSA) was applied. A coating was formed by diluting the MNP into 10 mL of Hexane (Biolab 110-54-3, 88.198 g.mol^−1^; Biolab, Ashkelong, Israel) and 50 mL of double distilled water. Aqueous DMSA 10% (M/V; Alfa Aesar A17909, 182.22 g.mol^−1^; Alfa Aesar, Yehud, Israel) and 20 mL of acetone were added after MNPs were homogeneously suspended. The suspension was first stirred for 48 h on a low frequency and then again 48 h at a high frequency at room temperature. Finally, hydrophilic soluble MNPs were separated from aggregates.

### Transmission electron microscope

MNPs were diluted 1000 × before mounting one drop of the MNP solution on a transmission electron microscopy (TEM) grid (Carbon Type A). After, they were left to dry for 48 h at room temperature.

Samples were imaged by TEM (DMi8; Leica Microsystems, Wetzlar, Germany) and a JEM-2010F (JEOL, Tokyo, Japan) equipped with a UHR pole piece, operated at 200 kV. Phase-contrast and bright-field diffraction images were recorded on a K2 Summit direct electron detector (Gatan-Ametek, Pleasanton, CA), connected to the microscope and set to linear mode.

### Fabrication of magnetic cantilevers

Preparation of magnetic cantilevers was based on a previously described technique.^23^ Briefly, by dissolving 100 mg of gelatin (Gelatin Type A, 175 bloom from porcine skin; Sigma-Aldrich, Milwaukee, WI) in 0.5 mL of Dulbecco's phosphate-buffered saline (PBS), a 20% (w/v) gelatin solution was prepared. The solution was mixed and placed in a 65°C water bath until completely dissolved. To prepare the 8% (w/v) active RM transglutaminase solution, 40 mg of transglutaminase powder (Active RM transglutaminase; Ajinomoto Corp, Tokyo, Japan) was dissolved in 0.5 mL of PBS. The solution was mixed and placed in a 37°C water bath until the transglutaminase was completely dissolved.

To fabricate the magnetic cantilevers, the gelatin and transglutaminase solutions were thoroughly mixed and dispensed into 3D-printed PLA (RaisePro; Raise Technologies, Inc., Chapel Hill, NC) molds clamped on a glass cover-slide. Then, 125 μL of the mixture was poured in each mold to form six 20 × 6 × 0.2 mm cantilevers per batch. Within 4 min after casting, polydimethylsiloxane (PDMS) stamps were placed on the still viscous gel solution. PDMS stamps were fabricated, as previously described,^23^ and presented line features with 5-μm-deep, 10-μm-wide grooves and 5-μm-wide ridges. After 24 h, the PDMS stamps were carefully detached, the PLA molds were lifted and removed, while the magnetic cantilevers remained on the glass slide. Cantilevers were cut to 6 mm wide using a scalpel. To detach the cantilevers, they were soaked in distilled water for 5 min and then were carefully scraped from the glass using a razor blade. Hydrogel cantilevers were stored at 2°C in a parafilm-sealed Petri dish.

### Characterization of magnetic cantilevers

#### Profilometer

Profilometer imaging was conducted using Olympus LEXT 4000 optical profilometer (Olympus Corporation, Tokyo, Japan). The magnetic cantilever was imaged to demonstrate the microgrooves created by patterning it with a PDMS stamp, as previously described. Collected data were processed, using MATLAB (V. 2020b).^24^

### Image analysis

An iPhone 12 was placed close to the setup so that the cantilever could be filmed from the side. Cantilevers were recorded with a frame rate of 30 fps. Videos were saved as .mp4 files and then converted to .avi. Videos were further processed to binary, wherefrom the x and y positioning of the cantilever tip were subtracted for each frame, using MATLAB (V. 2020b).^24^

### Human neuroblastoma SH-SY5Y cell cultures

Human neuroblastoma SH-SY5Y cells, infected with α-syn(A53T)/green fluorescent protein (GFP) adeno-associated virus, were provided by Prof. Uri Ashery's lab. Cells were grown in 1:1 RPMI/F12 (Satorius, Kibbutz Beit Haemek, Israel) medium supplemented with 10% fetal bovine serum (FBS; Sartorius), 0.15% sodium bicarbonate (Sartorius), 1% Glutamax (ThermoFisherScientific, Waltham, MA), 100 U/mL of penicillin, and 100 μg/mL of streptomycin (Sartorius), at 37°C with 5% CO_2_ in a humidifying incubator. For experiments, cells were differentiated to “neuron-shaped-like” as detailed in the “cell differentiation” part, which follows.

### SH-SY5Y cell differentiation

SH-SY5Y cells were differentiated in two phases over 11 days. First, magnetic cantilevers were coated with 10 mg/mL of poly-D-lysine (ThermoFisherScientific) and, subsequently, with 4 μg/mL of laminin (Sigma-Aldrich). Cells were then mixed 1:1 with Matrigel (354234; Corning Incorporated, Corning, NY) and seeded on the coated magnetic cantilevers (150,000 cells per cantilever) and allowed to grow for 24 h. In the first phase of differentiation, half of the medium was changed to medium containing additionally 10 μM of retinoic acid (Sigma-Aldrich). Medium was refreshed after 48 h. On day 8 of the differentiation, all the medium was changed to FBS-free medium containing 2 ng/mL of brain-derived neurotrophic factor (PeproTech, Cranbury, NJ) for the second phase. Differentiation was completed after 3 more days of incubation.

### Endothelial cell culture

Human endothelial cells EA.hy926 (ATCC CRL-2922; American Type Culture Collection, Manassas, VA) were used after stable lentiviral transfection of GFP (a kind gift from Dr. Shelly Loewenstein from Prof. Lahat's lab, Tel Aviv Sourasky Medical Center). Cells were cultured in Dulbecco's modified Eagle's medium high glucose (Satorius), supplemented with 10% FBS (Sartorius), 100 U/mL of penicillin, and 100 μg/mL of streptomycin (Sartorius), at 37°C with 5% CO_2_ in a humidifying incubator. Cells were seeded on the magnetic cantilevers (85,000 cells per cantilever) and used for experiments 24 h later.

### Cell viability assay

Cell viability was monitored using AlamarBlue Cell Viability Reagent (Invitrogen, ThermoFisherScientific). AlamarBlue is a resazurin-based, membrane-permeable assay in which the reduction of resazurin by living cells directly correlates with cell viability. The reagent was added into the media, according to manufacturer's instructions, and incubated for 3 h. The assay was performed before treatment for initial cell viability, 1 h after TBI induction and 24 h later. Fluorescent intensity was measured with the Tecan Spark multi-mode microplate reader and normalized to the blank and control samples. Cell viability was calculated by determining changes between the initial measured cell viability and at the two time points after TBI induction.

### Imaging (optical and epifluorescence)

Light microscopy images were taken by a Nikon Eclipse TS100 microscope (Nikon Corporation, Tokyo, Japan). Fluorescence images were captured using the Olympus IX83 inverted microscope and Olympus FV3000 confocal laser scanning microscope (Olympus Corporation) and were imaged with a 10 × or 20 × objective.

### Experimental setup

**FIG. 2.**
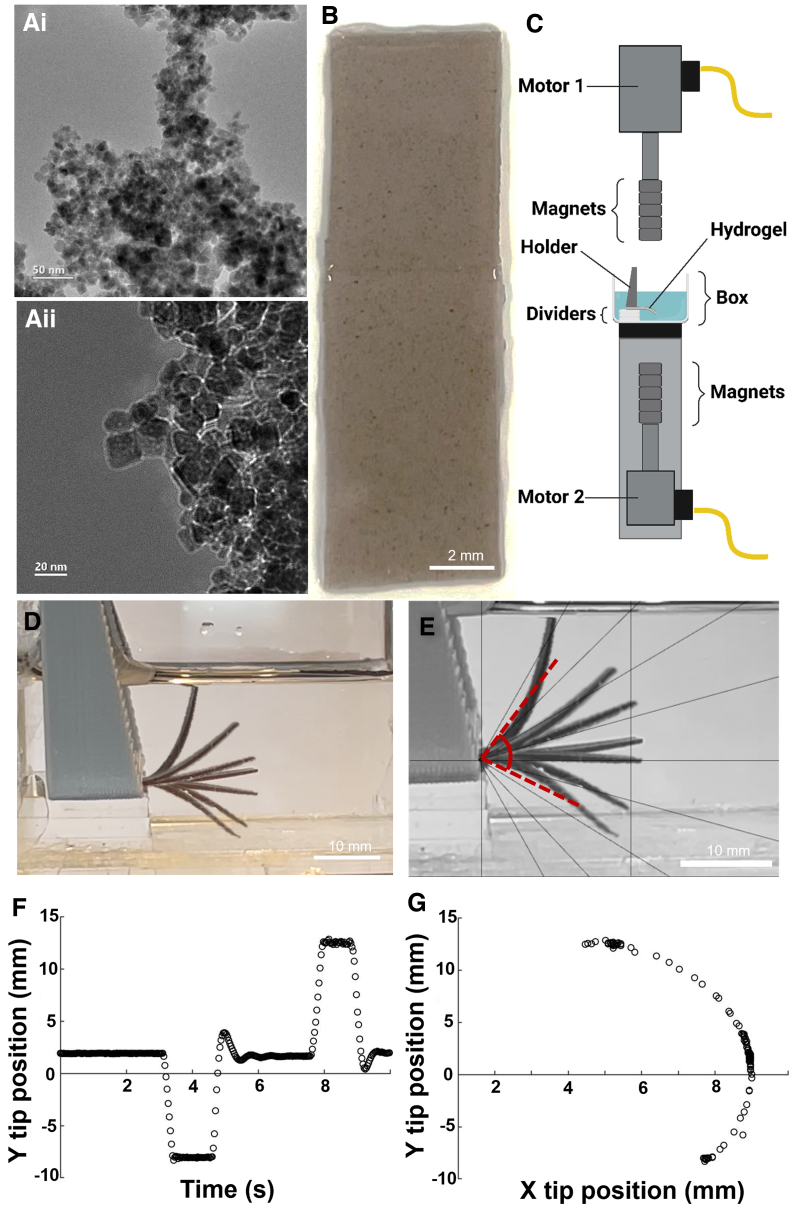
Experimental setup. (**Ai, ii**) Representative transmission electron microscope images of the MNPs. (**B**) Full-length gelatin-based hydrogel scaffold containing MNPs. (**C**) Side view of the platform setup, consisting of five magnets attached to linear motors, disposed on the top and under the box. When the magnet is moved to zero distance to the box, it has a 10-mm distance to the cantilever. (**D**) Reconstitution of cantilever bending as a response to the movement of the magnets. (**E**) Relative angle of cantilever movement. (**F**) Characterization of cantilever positions over time and (**G**) and correlated to the x-position. MNPs, magnetic nanoparticles.

The general concept is based on the platform that is described at Hagel and colleagues.^25^ A schematic of the experimental setup is shown in Figure 2C. The basic concept of the system consists of two stacks of permanent magnets attached to two linear motors (LinMot PS01-23x160-R; Fig. 2C, Supplementary Figs. S1 and S2), which, by moving toward the magnetic cantilever or away from it, control its actuation. The controller (C1100 controller, LinMot) is connected to a computer by a USB to an RS-485 converter adapter. The control software, written in Visual Studio (Microsoft Corporation, Redmond, WA), allows to modify the systems parameters. The experimental setup consists of a clear polymethyl methacrylate box (47 × 47 × 30 mm), which is positioned vertically centered between the two linear motors, and is filled with PBS. The cantilever is placed horizontally inside the box, using spacers, 8 mm above its floor (Fig. 2C, Supplementary Figs. S1 and S2).

To mimic the forces exerted on brain tissue during TBI, the cantilever was constrained such that a part of it was fixed by the apparatus, while the other part was free to move. The deformation was obtained through the interaction of the external magnets with the magnetic nanoparticles homogenously embedded in the cantilever. The linear motor moved the attached magnets at ∼10 m/s.

### Simulations

**FIG. 3.**
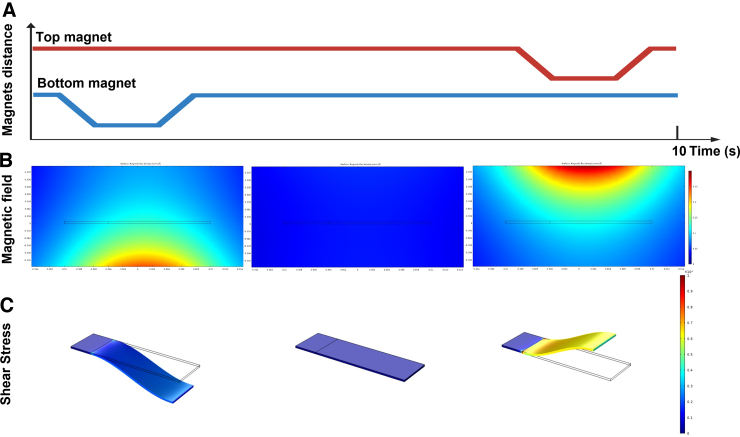
Exerted forces. (**A**) Illustration of magnets' distance to the cantilever over time. First, bottom magnets (blue) move closer to the cantilever, then move back to the initial position. Subsequently, top magnets (red) move closer to the cantilever and return to the starting position. (**B**) Representative simulation of the magnetic field around the cantilever in the different positions of the magnets. (**C**) Representative simulation of von Mises stress exerted on the cantilever attributable to its deformation under the magnetic field in the different positions.

The interaction of the magnetic field, originating from the stacks of permanent magnets, with the magnetic nanoparticles embedded in the elastic cantilever, as well as the movement and deformation resulting therefrom, were simulated using *COMSOL Multiphysics*.^26^ By utilizing the magnetic-fields module, solid-mechanics module, and moving-mesh module, we were able to reconstruct the dynamics observed in the experiment to within decent agreement. This allowed extraction of the magnetic flux density distribution in space and the forces acting on the gel, as well as the stresses developed in it, its movement and deformation, according to the movement of the magnets (Fig. 3). Simulations were run both in 2D as well as 3D, using first a stationary solver to obtain the steady (resting) state of the cantilever in the presence of the magnets (situated in their farthest positions), and feeding that solution to a time-dependent solver accounting for the dynamics while moving the magnets under the same movement profile captured and analyzed with the camera in the experiment. The physical parameters of the system were fine-tuned from their literature-based values until the simulations sufficiently converged with the experimentally observed movement values. A 3D simulation of the exact experimentally captured movement of the gel was also run to further investigate the stress, compressive and tensile forces acting on the gel, by forcing the movement profile without addressing the actuation mechanism (no magnetic fields and forces).

### Statistical analysis

All experiments were performed in at least three independent replicates. The results shown are presented as mean ± standard deviation (SD) from individual experiments. Using Graphpad Prism (V. 9.4.1; GraphPad Software Inc., La Jolla, CA) software, *p* values were calculated for differences between multiple groups by one- or two-way analysis of variance followed by Tukey's multiple comparison test. A statistically significant difference between two data sets was assessed, and *p* < 0.05 was considered statistically significant.

## Result

### Dynamic tissue-loading establishment

The goal of this study was to develop a model to comprehend the dynamics involved in TBI, with an emphasis on distinguishing between the tension and compression mechanisms of acceleration/deceleration injuries. The first step in establishing the dynamic tissue-loading (DTL) model was to create magneto-responsive hydrogel (Mgel) cantilevers that would support the growth of endothelial and neuron-like cells. To do so, we synthesized MNPs with an average diameter of 15–17 nm and incorporated them into a gelatin-based hydrogel matrix (Fig. 2Ai,Aii,B). A platform was then established to apply selective forces on Mgels that mimicked brain-like tissue, enabling to simulate the effects of whiplash injury by manipulating the movement of magnets in relation to the Mgels (Fig. 2C,D). The gel was able to respond to the movement of the magnets in ∼2 sec, and reach its maximum position, up to 13 mm and down to 8 mm from the initial position (0 mm; Fig. 2F,G). Placement of magnet 5 above and below the gel induced a deformation angle of the Mgel of 54.5 and 24.5 degrees for, respectively, the top and down magnet movement (Fig. 2E).

Figure 3 shows the simulated field landscape in the vertical plane. The dynamic chance of the field is correlated with the changes in the shear that is applied on the gels and tissues. Moreover, Figure 3B demonstrates that the magnetic field is equally distributed on ∼85% of the tissue and the field decays in ∼20%**.** The peak acceleration simulated because of the magnetic fields, measuring up to ∼0.125 T inside the gel, was ∼0.17 G (compared to ∼0.2 G in the experiment), with forces in the range of 50 μN. This suggests that the experimental apparatus measures mostly the effects of compressive and tensile forces and deformations acting on the cells, while decoupling brute-force mechanical trauma, given that all actuation is contactless. The deformation in the full simulation probes von Mises stresses ranging from 150 Pa (at the resting position) to 1500 Pa (at the most deformed position). The purely mechanical simulation of the experimentally captured movement, though less accurate, puts the maximum stress closer to 50 kPa.

### Cellular integration

**FIG. 4.**
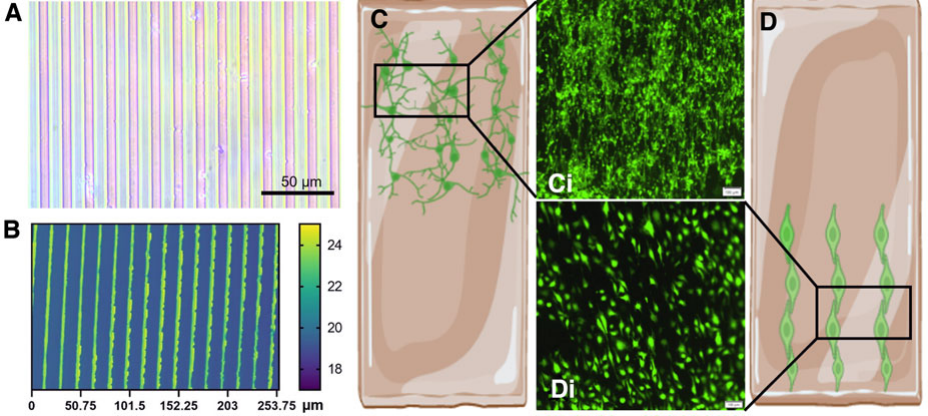
Depositing cells on the magnetic cantilevers. (**A**) Optical micrograph of the micropatterning stamp, with a pattern line of 10 μm wide with a spacing of 5 μm. (**B**) Topography of the stamped groove pattern on the scaffold. (**C,D**) Illustration of micropattern hydrogels with (**C,Ci**) aligned differentiated SH-SY5Y cells and (**D,Di**) endothelial cells. Scale of the confocal imaging, 100 μm.

**FIG. 5.**
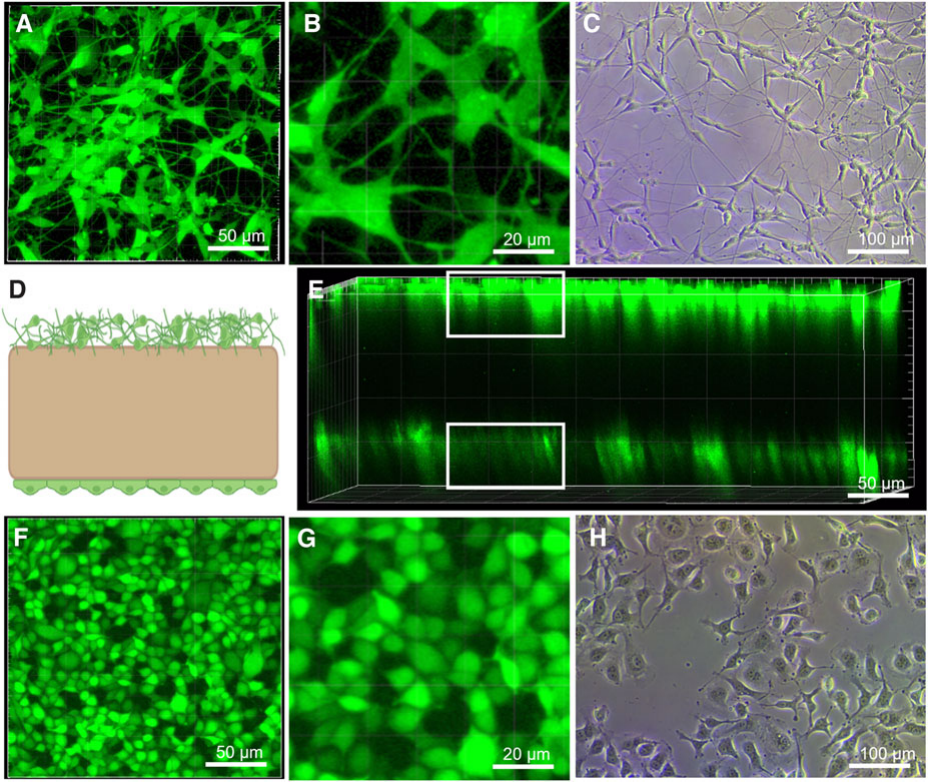
Coculture of endothelial and neuron-line cells on Mgels. (**A)** Representative confocal microscope image of differentiated SH-SY5Y cells on magnetic cantilevers (**B**), zoom of (A). Comparison to (**C**) brightfield microscope image of differentiated SH-SY5Y cell morphology, grown in a Petri dish. (**D**) Illustration of magnetic cantilevers with neurons on top and endothelial cells in the bottom. (**E**) 3D reconstitution from Z-stack confocal microscopy of the magnetic cantilevers with SH-SY5Y on the top face and endothelial cells on the bottom face. (**F**) Representative confocal microscope image of endothelial cells on magnetic cantilevers (**G**), zoom of (F). Comparison to (**H**) brightfield microscope image of endothelial cell morphology, grown in a Petri dish. Mgels, magneto-responsive hydrogels.

After computational simulation of the DTL platform, the suitability of the model to induce TBI *in vitro* was investigated. The top surface of the gel was micropatterned with microgrooves of 10 μm width and 5 μm depth (Fig. 4A,B) in order to achieve parallel cellular alignment, which may improve cellular functionality and ensures equal force distribution, applied in length onto the neurons^27^ (Fig. 4C,D, Supplementary Fig. S4). To test biocompatibility of the platform, neuron-like differentiated neuroblastoma cells (SH-SY5Y) and endothelial cells were used. Cells were successfully cultured on the Mgels (Fig. 5A,B,F,G) and exhibited a similar morphology to those grown on a Petri dish (Fig. 5C,H; Supplementary Fig. S3). The Mgel also enabled the coculture of cells on both sides of the cantilever. Here, with neuronal cells on the top and endothelial cells on the bottom (Figs. 4C,D and 5), separated by a 300-μm-thick gel, the thickness of the gel can be modified to adjust the distance between the cells.

### Cellular response to mechanical perturbation

After we validated that the Mgel can support the cellular viability of multiple cell types (endothelium and neurons), we identified the cellular response to the DTL (e.g., compression, tension, and compression + tension [full TBI]). To do so, we characterized cellular viability, number of cells, and morphology.

**FIG. 6.**
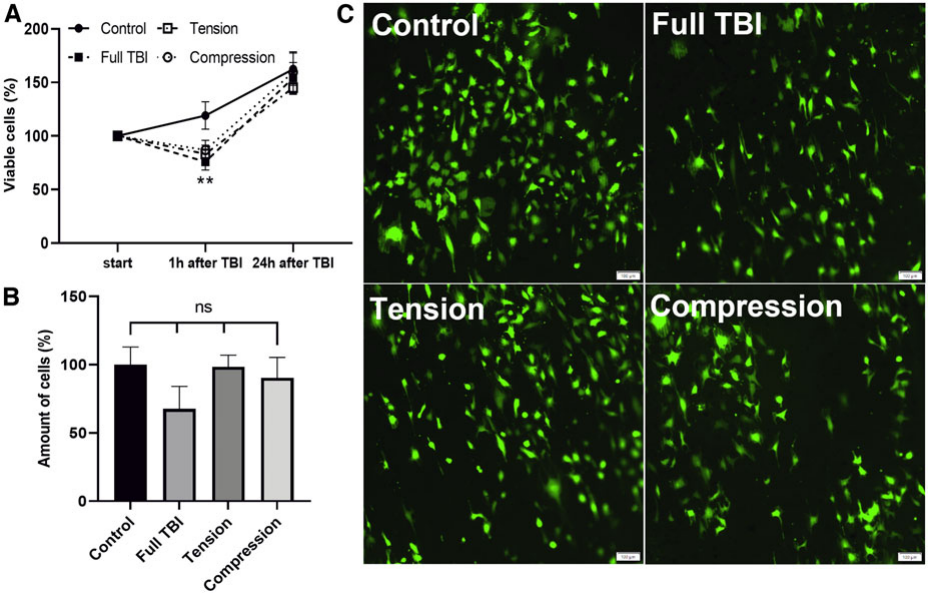
Endothelial response to force induction. (**A**) Viability quantification overtime in percentage of initially determined cell viability. (**B**) Quantification of cells in percentage relative to control 24 h after force induction. (**C**) Epifluorescent microscope images of endothelial cells 24 h after TBI: control, full TBI (compression + tension), tension, and compression. Scale, 100 μm. The results shown are presented as mean ± SD from individual experiments. SD, standard deviation; TBI, traumatic brain injury.

As shown in Figure 6, endothelial cells respond to force induction (compression, tension, and compression + tension [full TBI]) in the parameters that were used (Fig. 3), and that the force induction significantly reduces cell viability 1 h after induction (Fig. 6A). Whereas the control continued to grow, viability for TBI samples dropped to ∼70% of the initially determined viability. However, no significant differences between the forces applied were observed. After 24 h, endothelial cells recovered and started to proliferate again with the total amount of cells comparable to the control sample (Fig. 6B). Moreover, there were no changes in morphology of the endothelial cells observed (Fig. 6C).

**FIG. 7.**
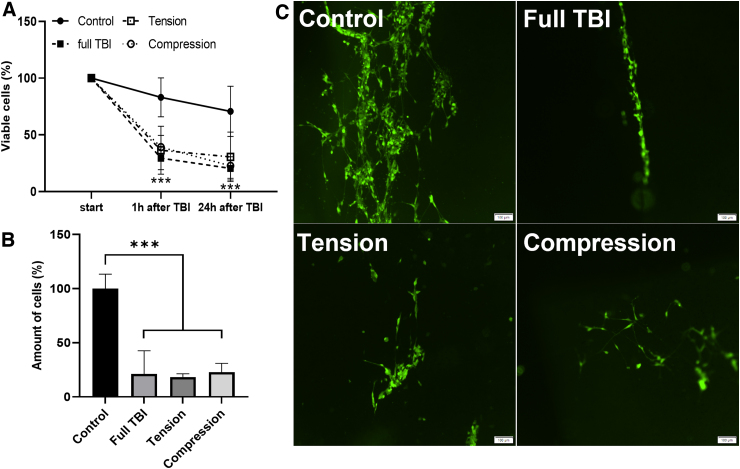
Behavior of neuron-like SH-SY5Y cells on cantilever bending. (**A**) Viability quantification overtime in percentage of initially determined cell viability. (**B**) Quantification of cells in percentage relative to control 24 h after force induction. (**C**) Epifluorescent microscope images of SH-SY5Y cells 24 h after TBI: control, full TBI (compression + tension), tension, and compression. Scale, 100 μm. The results shown are presented as mean ± SD from individual experiments. SD, standard deviation; TBI, traumatic brain injury.

In contrast to endothelial cells, DTL causes an irreversible effect on the neuronal culture. As shown in Figure 7A, 1 h after force induction, cellular viability decreased to ∼40% of the initial viability and continued to drop to ∼30% over the following 24 h, as observed in the images taken after 24 h (Fig. 7B,C).

Similar to the results observed for endothelial cells, the application of compression, tension, or full TBI (compression + tension) did not cause significantly different responses in neuronal cultures.

## Discussion

Previous studies^18^ demonstrated that rapid angular/rotational acceleration-deceleration injuries consistently evoked physiological responses that were paralleled by pathological changes observed throughout the subcortical white matter in contusion models on fixed heads. These mechanical forces also lead to the release of chemicals that further contribute to the development of oxidative stress and inflammation, damaging brain cells. One of the challenges in studying mechanobiology is to understand how those mechanical forces contribute to the development and progression of brain damage. To address this challenge, we developed a dynamic tissue loading system that allowed us to analyze the various effects of the fundamental components of acceleration-deceleration injury. This enables us to further understand the mechanisms underlying this complex condition. Though TBI can range in severity and the forces applied to the brain tissue up to 1000 kPa in severe cases,^28^ the dynamic tissue loading system is modular and can simulate TBI with different acceleration rates and shear intensities, by controlling the speed of the motor, geometry of the magnetic cantilever, and distance of the magnets from the magnetic cantilevers.

In this study, shear stresses up to 50 kPa were used, which are in the range of shear values applied during a mild TBI.^29^

The platform's ability to separate the processes of mechanical force (compression and tension) while tracking the cellular response enables the investigation of various mechanobiological responses to TBI. One advantage of the platform is the use of gels as a cell growth substrate, which more closely mimics the natural cellular microenvironment. Stiffness of the gels is significantly softer than the glass or plastic commonly used in neuronal cultures. Gels also allow for the culture of cells in a 3D environment or on the surface of the gel (as is the case in this study). Additionally, gels enable the use of many traditional biological tools for cellular analysis, such as fluorescents assays, immunohistochemistry, transcriptomics, etc., but may not be suitable for functional assays like transepithelial electrical resistance or multi-electrode array recording.

Another challenge, which is relevant for many *in vitro* TBI models, is the heterogenous injury induced to the cells on the gels. The cells in the middle of the gel will experience a stronger deformation than the cells sitting at the tip of the gel. These differences in degree of injury must be taken into account during quantification of cell injury. In perspective, to overcome this problem, it would be possible to use shorter gels to reduce the variability of induced injury.

A possible next step for the experimental platform presented here, which displays primarily the effects of tensile and compressive dynamics during deformation, is modifications that will address the extreme opposite end of the scale, where deformation of the gel will be minimal, but acceleration of the cells will be sudden and more significant—again without any external mechanical contact. Having the capability to probe, separately and controllably, damage attributable to deformation, and attributable to sudden acceleration, can dramatically enhance our ability to understand the mechanisms of TBI. Further steps for the experimental platform will be to expose the cultures to novel drugs in the search for effective pharmacological strategies.

The aim of this study was to develop a modular platform that allows the application of separate mechanical stimuli, including the compression and tension that induce diffuse axonal injury, in order to better understand the response of endothelial and neuronal cells to these forces after TBI. We found a significant decrease in viability for both cell types 1 h after the application of mechanical force. However, no significant differences were observed between the application of tension, compression, or both together. This may be attributable to a number of factors, including the moderate shear forces applied during the cantilever contraction, which are similar to those experienced in mild TBI, and the sensitivity of both neuronal and endothelial cells to mechanical perturbation, which has been previously demonstrated in the literature.^30,31^ In contrast to neurons, endothelial cells have the ability to proliferate, as shown 24 h after injury, when the total number of cells was similar to the control sample. These findings highlight the differences in the response of these two cell types to injury, including their ability to recover, the number of live cells, and morphological changes, which is consistent with previous research on the cellular response of endothelial and neuronal cells to injury.^32,33^

In conclusion, we have established a DTL platform suitable to separate the mechanical forces in TBI. Using the capabilities of this novel model system, under different conditions (such as intensities, acceleration rates, etc.), will be beneficial in better understanding the mechanobiology and cellular responses post-TBI and contribute to the ongoing attempts to bring promising therapies into clinical practice.

## Supplementary Material

Supplemental data

Supplemental data

Supplemental data

Supplemental data

## Data Availability

The data that support the findings of this study are available from the corresponding author upon reasonable request.

## Transparency, Rigor, and Reproducibility Summary

This study was not formally registered because the article does not contain clinical studies or patient data. The analysis plan was not formally pre-registered, but the team member with primary responsibility for the analysis (corresponding author) certifies that the analysis plan was pre-specified. A sample size of at least three independent cell cultures was planned and conducted. Handling of biomechanical data was performed by team members who were aware of relevant characteristics of the samples. All data were acquired in the same time frame and with the same devices. Authors are aware of the limitations of the assays used, and assay-specific limitations can be found on manufacturer websites. All equipment and analytical reagents used to perform the biomechanical measurements are widely available from companies. The statistical tests used were based on the assumptions of normal distributions, and the sample sizes and degrees of freedom reflect the number of independent measurements. Methods that do not require correction for multiple comparisons were used. No replication or external validation studies have been performed or are planned/ongoing at this time to our knowledge.

The data that support the findings of this study are available from the corresponding author upon reasonable request. There is no analytical code associated with this study. All materials used to conduct the study were obtained from a widely available source, as stated in the Methods section. The authors agree or have agreed to publish the manuscript using the Mary Ann Liebert Inc. “Open Access” option under the appropriate license.

## Abbreviations Used

DAI: diffuse axonal injury
DMSA: meso-2,3-dimercaptosuccinic acid
DTL: dynamic tissue-loading platform
FBS: fetal bovine serum
GFP: green fluorescent protein
Mgel: magneto-responsive hydrogel
MNPs: magnetic nanoparticles
PBS: phosphate-buffered saline
PDMS: polydimethylsiloxane
SD: standard deviation
TBI: traumatic brain injury
TEM: transmission electron microscopy
