# Supplementary material for: Inducing Mechanical Stimuli to Tissues Grown on a Magnetic Gel Allows Deconvoluting the Forces Leading to Traumatic Brain Injury
Source: Neurotrauma Rep. 2023 Aug 23;4(1):560–72. doi: 10.1089/neur.2023.0026 (PMC10457614; doi:10.1089/neur.2023.0026)
Supplement: Supplemental data [file Suppl_FigureS2.docx]

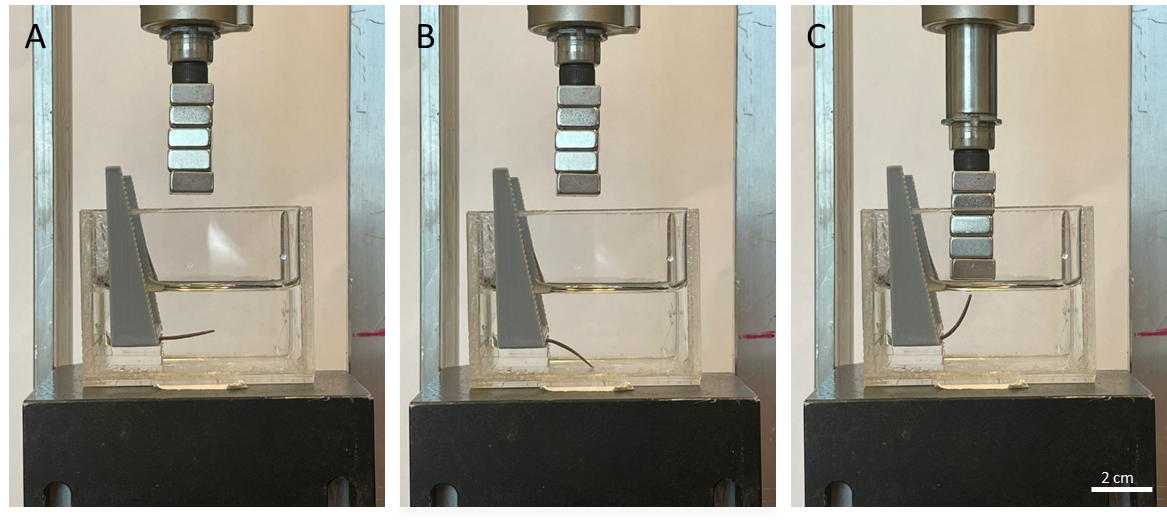


SI Fig. 2: Magnetic cantilever max. positions **(A)** neutral **(B)** bottom magnets up – cantilever bends down **(C)** top magnets down – cantilever bends up.
