# Supplementary material for: Inducing Mechanical Stimuli to Tissues Grown on a Magnetic Gel Allows Deconvoluting the Forces Leading to Traumatic Brain Injury
Source: Neurotrauma Rep. 2023 Aug 23;4(1):560–72. doi: 10.1089/neur.2023.0026 (PMC10457614; doi:10.1089/neur.2023.0026)
Supplement: Supplemental data [file Suppl_FigureS4.docx]

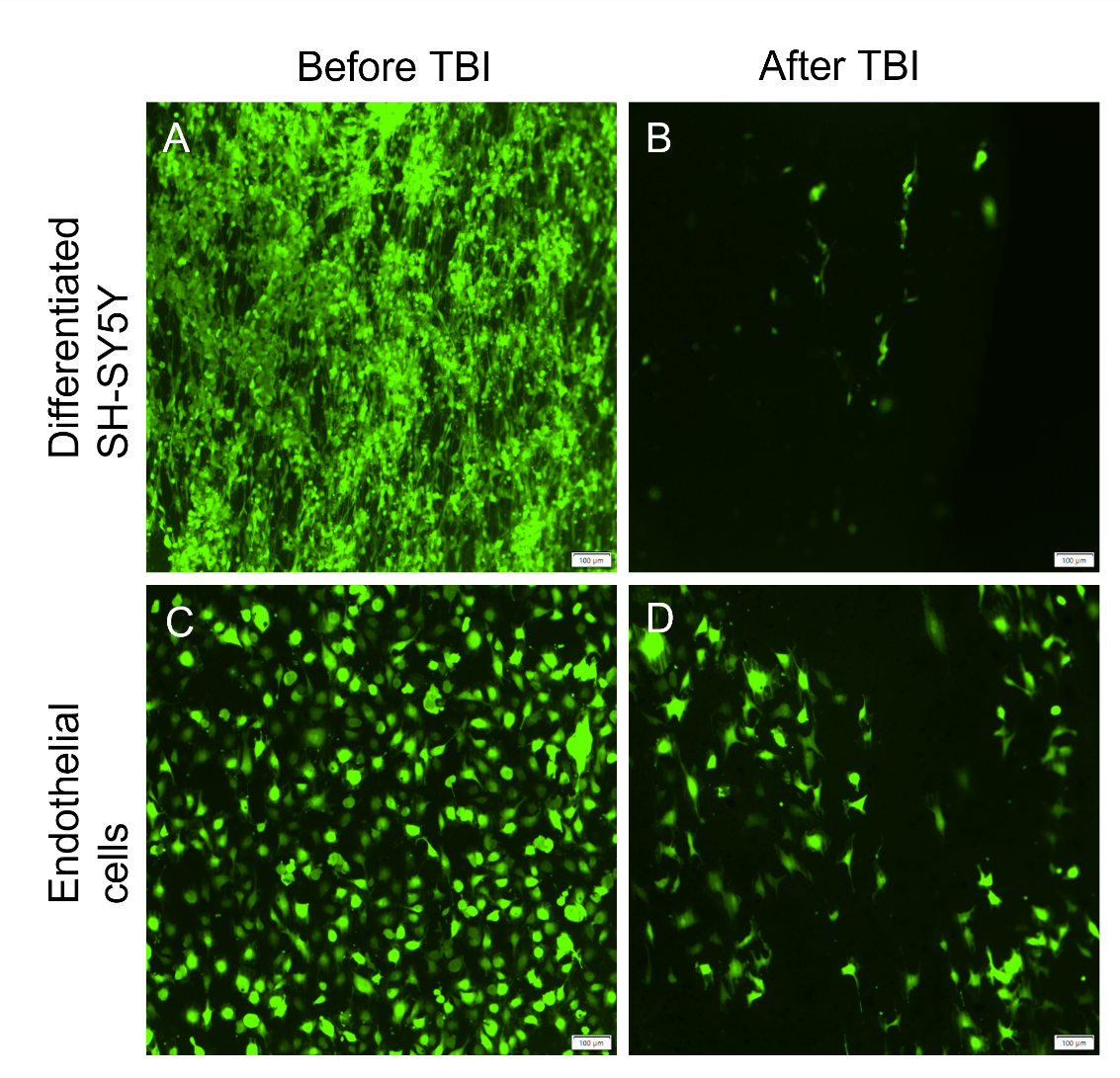


SI Fig. 4: Effect of gel micropattern on cell growth. **(A, B)** differentiated SH-SY5Y cells and **(C, D)** endothelial cells display cell orientation along the micropattern. **(A, C)** before TBI and **(B, D)** after TBI.
