# Supplementary material for: Inducing Mechanical Stimuli to Tissues Grown on a Magnetic Gel Allows Deconvoluting the Forces Leading to Traumatic Brain Injury
Source: Neurotrauma Rep. 2023 Aug 23;4(1):560–72. doi: 10.1089/neur.2023.0026 (PMC10457614; doi:10.1089/neur.2023.0026)
Supplement: Supplemental data [file Suppl_FigureS3.docx]

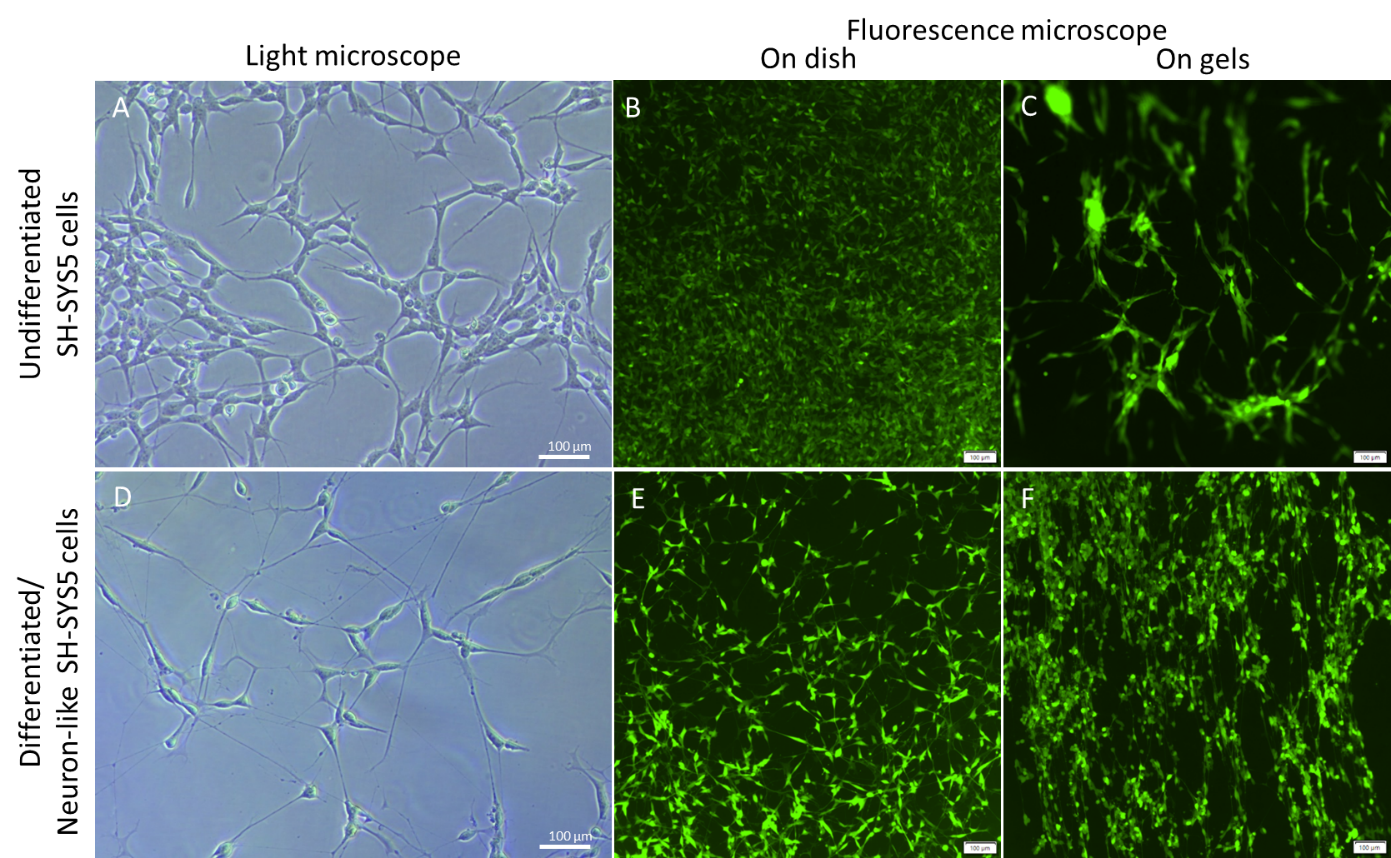


SI Fig 3: Morphology of SH-SYS5 cells before **(A,B,C)** and after differentiation **(D,E,F)**. Images taken with a light microscope **(A, D)** and fluorescence microscope **(B,C,E,F)**. **(A,B,D,E)** show morphology on a regular plastic dish and **(C,F)** of cells grown on magnetic cantilevers.
